# Microsurgical Reconstruction of Complex Scalp Defects With Vastus Lateralis Free Flap

**DOI:** 10.1002/micr.70025

**Published:** 2025-01-29

**Authors:** Giovanni Zabbia, Emanuele Cammarata, Mara Franza, Francesca Toia, Greta Tondini, Francesca Graziano, Domenico Gerardo Iacopino, Adriana Cordova

**Affiliations:** ^1^ Plastic and Reconstructive Surgery, Department of Precision Medicine in Medical, Surgical and Critical Care (Me.Pre.C.C.) University of Palermo Palermo Italy; ^2^ Neurosurgery Unit, Department of Head and Neck Surgery Garibaldi Hospital Catania Italy; ^3^ Neurosurgical Clinic AOUP “Paolo Giaccone”, Post Graduate Residency Program in Neurologic Surgery, Department of Biomedicine Neurosciences and Advanced Diagnostics, School of Medicine University of Palermo Palermo Italy

**Keywords:** microsurgical reconstruction, scalp reconstruction, soft tissue reconstruction, vastus lateralis free flap

## Abstract

**Background:**

Scalp reconstruction is a challenging field for plastic surgeons. In case of large or complex defects, microsurgical‐free flaps are usually required. Reconstructive failure can result in high morbidity and in some cases be life‐threatening.

In this article, we present our experience in the reconstruction of complex scalp defects with the use of a vastus lateralis (VL) free flap.

**Patients and Methods:**

From July 2013 to July 2023, we retrospectively analyzed patients who underwent soft tissue reconstruction of the scalp with a VL‐free flap at the authors' institution. The patient's demographic, clinical and surgical characteristics, and postoperative complications were recorded and analyzed.

**Results:**

Thirty patients were included. The mean age was 67.3 years. Seventeen patients were male, while 13 were female. In 56.7% of patients, defects resulted from cancer resection. In four patients, the defect was limited to the soft tissues while a multi‐layer defect with bone and/or dura involvement was present in 26 patients. Soft tissue reconstruction was always achieved with a VL‐free flap (*n* = 30). In 28 cases, a skin‐grafted muscular flap was used. The most used recipient vessels were the superior thyroid vessels (*n* = 18). Complications occurred in six patients (20%): two cases of total flap loss and two cases of infection of the cranioplasty materials requiring their removal. In two cases patients died within 48 h. All patients were satisfied with the aesthetic and functional results at 6 months.

**Conclusions:**

In the case of complex scalp defects, the gold standard is reconstruction through microsurgical flaps that provide well‐vascularized tissue and allow to cover large defects, reducing the incidence of infections and ensuring good brain protection even without cranioplasty. In our experience, VL‐free flap represents a valid option, providing a low donor site morbidity, the possibility of a two‐team approach, and a low complication rate.

## 1 Introduction

Scalp defects are usually the result of traumatic injuries, tumor excision, or complications of neurosurgical procedures such as infection of cranioplasty materials or bone necrosis. Such defects can involve only the soft tissues of the scalp or include the bone and/or the meninges. Reconstruction of these defects is a challenging field for plastic surgeons due to the complex anatomy of the region and the possible need to cover the delicate intracranial structures.

Available reconstructive techniques for the reconstruction of soft tissue defects of the scalp comprehend skin grafts, pedicled flaps, and free flaps. Skin grafts are adequate in case of partial‐thickness soft tissue defects, but they don't provide a feasible coverage in case of full‐thickness soft tissue defects or multi‐layer defects involving the underlying structures.

In these cases, transfer of well‐vascularized tissues, with or without additional bone and dural reconstruction, is required to achieve a reliable morpho‐functional reconstruction (Mehrara, Disa, and Pusic 2006) by ensuring an appropriate coverage of the soft tissue defect, an adequate protection of the encephalic structures and an acceptable long‐term aesthetic result. Failure of reconstruction can lead to high morbidity and sometimes can be life‐threatening (Afifi et al. 2010).

Microsurgical tissue transplant is required for large skin defects not suitable for reconstruction with local skin flaps or smaller defects including meninges and bone due to the need to provide a thicker coverage with a bulky muscle flap and prevent cerebrospinal fluid fistulas (Sosin et al. 2015).

The most common microsurgical flaps described for complex scalp reconstruction are the latissimus dorsi (LD) flap, the transverse rectus abdominis myocutaneous (TRAM) flap, the anterolateral thigh (ALT) flap, and the radial forearm flap (Afifi et al. 2010; Sosin et al. 2015; Lachica 2011; Hussussian and Reece 2002), with the former being considered the most popular one. Conversely, although the vastus lateralis (VL) free flap is largely described in the literature for head and neck reconstruction, it is rarely reported for scalp reconstruction (Lachica 2011).

In this article, we present our experience in the reconstruction of complex scalp defects with the use of a VL‐free flap, discussing its indications, advantages and potential drawbacks and evaluating its feasibility, according to the application of our reconstructive algorithm.

## 2 Patients and Methods

In this study, we performed a retrospective review of patients who underwent microsurgical scalp reconstruction with a VL flap at the author's institution from July 2013 to July 2023.

Inclusion criteria were: age > 18 years, reconstruction with a muscular or musculocutaneous VL free flap.

Patients who deliberately abandoned the follow‐up were excluded from the study.

Personal data regarding age, sex, ASA class, etiology of the defect, involved tissues (skin and soft tissues, bone, dura), size and location of the defect, surgical timing of reconstruction (immediate/delayed), type of flap (muscle‐sparing, muscular, musculocutaneous or combined perforator‐muscle flap), recipient's vessels, need for vein graft interposition, need for bone and/or dura mater reconstruction, postoperative complications (partial or total flap loss/reconstructive failure, soft tissue or prosthetic infection, seroma, hematoma, wound dehiscence, cerebrospinal fluid leak, meningeal or bone exposure, donor site complications), duration of hospital stay and length of postoperative follow‐up were collected and analyzed for each patient.

Tissue defects were classified as single‐layer or multi‐layer defects, depending on the involvement of soft tissues alone or in combination with deep structures such as bone and meninges, respectively. The preoperative instrumental assessment was carried out through a contrast‐enhanced CT scan with additional three‐dimensional integration that was especially useful in the planning of the reconstruction of multi‐layer defects. Clinical pictures were collected preoperatively and at 6 and 12 months postoperatively. Cosmetic outcome was assessed postoperatively through the evaluation of the flap's texture, color, and trophism at the recipient site and the aspect of the scar at the donor site. The functional outcome of the lower limb was evaluated preoperatively and 6 months after surgery through the Lower Extremity Functional Scale (Binkley et al. 1999).

### 2.1 Surgical Technique

In our series, all reconstructions were carried out through a microsurgical VL flap. In the case of the segmental muscle‐sparing approach, as described in the anatomical study of Toia et al. (Toia et al. 2015), the superficial partition of the VL muscle was harvested independently from the intermediate and deep ones. The technique started as a classical VL flap by isolating the pedicle along the medial border of the muscle and progressively following it to the source vessels. At this point, the dissection continued by elevating distally the superficial partition from the deep muscular aponeurosis that covers the intermediate partition and progressively following a major branch of the descending branch of the lateral circumflex femoral artery, that runs in the soft tissue layer separating the superficial and intermediate partitions and selectively supplies the superficial partition. Finally, the dissection was concluded by incising the superficial partition proximally, thus leaving it attached only by the pedicle.

When a combined muscle and perforator flap—or “Razor flap,” as described by Cavadas and Teran‐Saavedra (Cavadas and Teran‐Saavedra 2002)—was employed to increase the covering area of the flap, the dissection began as a standard ALT flap, with the skin paddle centered over a preoperatively marked musculocutaneous perforator. After perforator identification, the vessel was followed intramuscularly for a short course, long enough to allow independent rotation of the skin island over the underlying VL muscle. Therefore, the flap harvest proceeded as classically described for the VL‐free flap.

### 2.2 Statistical Analysis

Descriptive statistics were carried out through mean and range for continuous variables and frequency and percentage for categorical variables. Paired samples T‐test was used to compare the means of pre‐ and post‐operative LEFS scores. Statistical significance was set at *p* < 0.05.

## 3 Results

Thirty patients were included in the study. The mean age was 67.3 years (range 27–88). Seventeen patients (56.7%) were male, while 13 (43.3%) were female. ASA score was ≥ III in 80% of cases (24 out of 30). In 56.7% of cases (17 out of 30), patients underwent reconstructive surgery after oncological resection. The most common histological type was squamous cell carcinoma (SCC) (*n* = 13), followed by basal cell carcinoma (BCC) (*n* = 1), dermatofibrosarcoma protuberans (DFSP) (*n* = 1), eccrine adenocarcinoma (*n* = 1) and angiosarcoma (*n* = 1). Other causes of scalp defects were complications of previous neurosurgical procedures (*n* = 12) and trauma (*n* = 1).

In four patients, the defect was limited to the soft tissues (1 BCC, 1 SCC, 1 DFSP, and 1 neurosurgical complication). A multi‐layer defect was present in 26 patients: bone was involved in 14 cases of cancer and 12 cases of neurosurgical complications, while dura was also involved in two cases of cancer and two cases of neurosurgical complications, respectively. The mean size of the soft tissue defect was 96.9 cm^2^ (range 24–600).

Fifty percent of reconstructions were performed immediately, while the remaining ones were delayed in a secondary surgery. In nine cases (30%) the defect was selectively located in the parietal region and four cases (13.3%) selectively in the occipital region. In the majority of cases, we treated complex defects involving multiple scalp areas: five cases of parieto‐occipital defects (16.7%), 5 cases of parieto‐temporal defects (16.7%), 4 cases of fronto‐temporo‐parietal defects (13.3%) and three cases of temporo‐parieto‐occipital defects (10%). We had no cases of complex defects selectively located in the frontal or temporal region.

**FIGURE 1:**
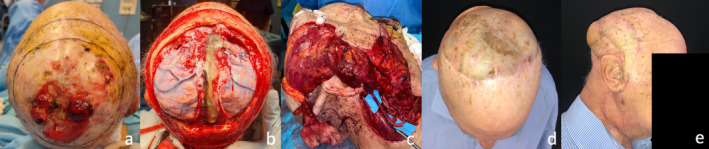
Clinical case of a 65 male patient who underwent scalp demolition for an angiosarcoma of the parieto‐occipital region with bone and dural invasion; (a) preoperative picture, (b) scalp defect after tumor excision, (c) reconstruction with vastus lateralis free flap covered with a split‐thickness skin graft, directly positioned on the dural substitute and anastomosed with the superior thyroid artery, and (d, e) postoperative result at 6 months without bone reconstruction.

**FIGURE 2:**
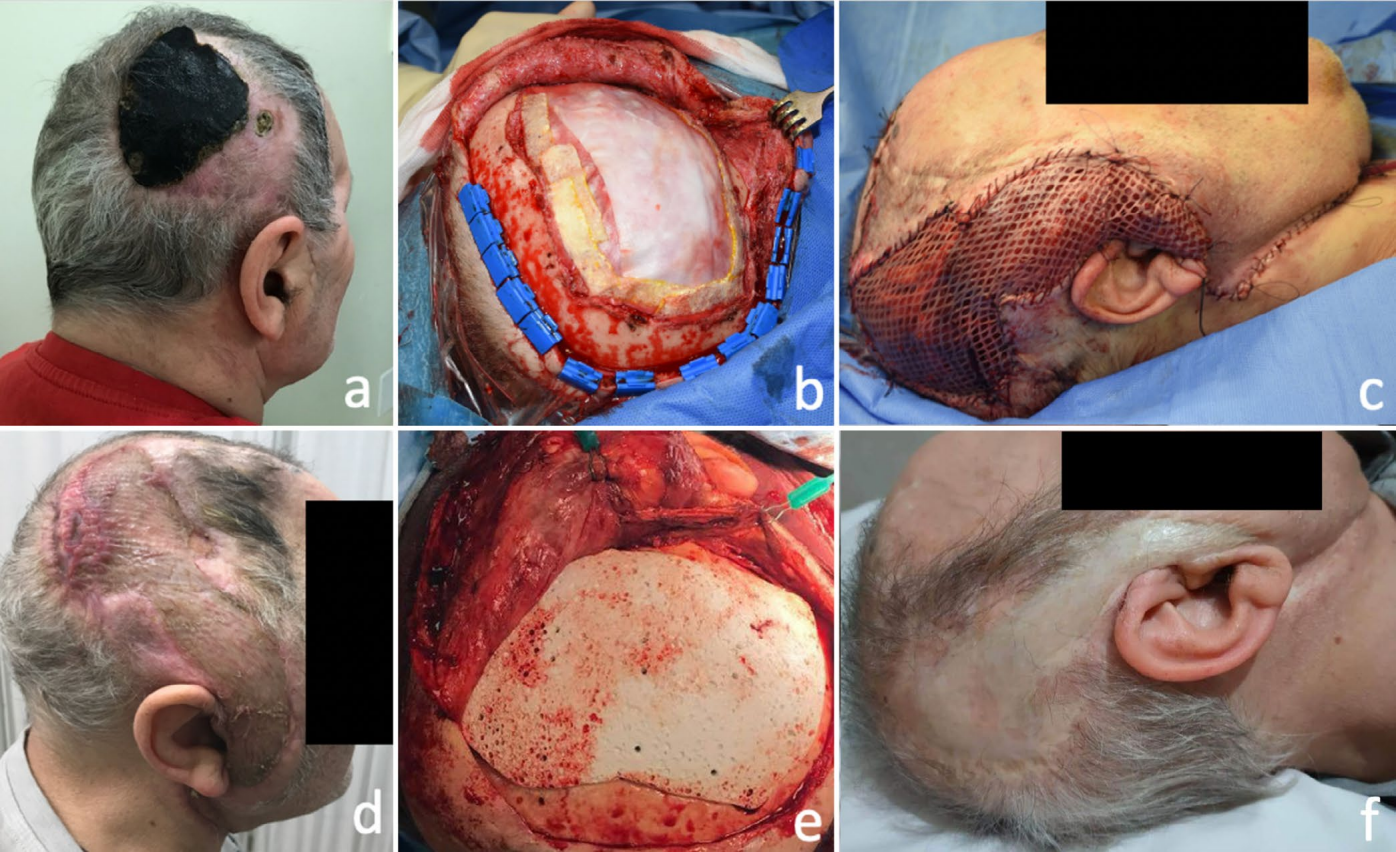
Clinical case of a 63 male patient with a necrotic eschar resulting from an exposition and infection of a hydroxyapatite custom‐made prosthesis after a decompressive craniectomy; (a) preoperative picture, (b) scalp defect after the removal of the infected cranioplasty material and the dural substitute, (c) reconstruction with skin‐grafted vastus lateralis free flap anastomosed with the superior thyroid artery and directly positioned on the dural substitute, (d) postoperative result at 3 months without bone reconstruction, (e) delayed calvarial bone reconstruction with hydroxyapatite implant, and (f) postoperative result at 2 years.

Soft tissue reconstruction was always performed with a VL‐free flap. In the majority of cases (*n* = 28), a skin‐grafted muscular flap was used for reconstruction (Figures 1 and 2). In 10 of these cases, the flap was raised with a muscle‐sparing approach, by harvesting only the superficial partition of the muscle as described by Toial et al. (2015). A musculocutaneous flap was employed in one patient; in one case, the flap was harvested as a combined perforator and muscular flap by including the skin paddle of the conventional ALT flap, that was freely rotated on its supplying perforator over the muscle (“Razor” flap) (Cavadas and Teran‐Saavedra 2002), increase the surface coverage. The mean size of the harvested muscle (length × width) was 14.5 (range 8–34) × 9.6 cm (range 6–16), with a mean surface of 93.1 cm^2^ (range 48–544).

The most used recipient vessels were the superior thyroid vessels (*n* = 18), followed by the superficial temporal vessels (*n* = 6) and the facial vessels (*n* = 6). In three cases, an interposition vein graft, harvested from the great saphenous vein, was required to lengthen the flap's pedicle.

The mean hospitalization period was 10 days (range 8–15). The mean preoperative LEFS score was 64.2 while the mean postoperative score was 63.6, with a non‐significant difference between pre‐ and post‐operative functional values (*p* > 0.05). In all patients, we observed a good color and texture match of the flap with a considerable reduction in muscle bulkiness and no pathologic scars at the donor site in the thigh at 6 months. Detailed characteristics of the patient sample are shown in Table 1.

**TABLE 1:** Characteristics of the patient sample.

Variable	*n* = 30
Age (years) (mean, range)	67.3 (27–88)
Sex (no, %)	
Male	17 (56.7%)
Female	13 (43.3%)
ASA score (no, %)	
<III	6 (20.0%)
≥III	24 (80.0%)
Etiology of the defect (no, %)	
Cancer	17 (56.7%)
Trauma	1 (3.3%)
Complications of neurosurgical procedure	12 (40.0%)
Size of the defect (cm^2^) (mean, range)	96.9 (24–600)
Location of the defect (no, %)	
Frontal	0 (0%)
Temporal	0 (0%)
Parietal	9 (30%)
Occipital	4 (13.3%)
Parieto‐temporal	5 (16.7%)
Fronto‐Temporo‐parietal	4 (13.3%)
Parieto‐occiptal	5 (16.7%)
Temporo‐parieto occipital	3 (10%)
Type of defect (no, %)	
Single‐layer	4 (13.3%)
Multi‐layer	26 (86.7%)
Bone involvement	26 (86.7%)
Dural involvement	4 (13.3%)
Timing of soft tissue reconstruction (no, %)	
Immediate	15 (50.0%)
Delayed	15 (50.0%)
Type of vastus lateralis flap	
Skin‐grafted muscular flap	28 (93.4%)
Musculocutaneous flap	1 (3.3%)
Combined perforator and muscular flap (Razor)	1 (3.3%)
Size of vastus lateralis flap (cm^2^) (mean, range)	93.1 (48–544)
Recipient vessels	
Superficial temporal vessels	6 (20.0%)
Facial vessels	6 (20.0%)
Superior thyroid vessels	18 (60.0%)
Need for interposition vein graft (no, %)	
Yes	3 (10.0%)
No	27 (90.0%)
Length of hospitalization (days) (mean, range)	10.0 (8–15)
Length of follow‐up (months) (mean, range)	32.0 (12–60)

Postoperative complications occurred in six patients (20%). We observed four cases of complete flap loss (13.3%) in the early postoperative period (within 48 h): in one case we performed a new reconstruction with a contralateral VL‐free flap, in one case a dermal substitute was used as a second‐choice salvage reconstruction while two patients were not suitable for secondary major surgery and died in the Intensive Care Unit in the early postoperative days. No cases of hematoma or seroma occurred in the immediate postsurgical period. No leakage of cerebrospinal fluid (CSF) or exposure of the underlying structures occurred in the long‐term period. The mean duration of follow‐up was 32 months (range 12–60 months).

A summary of postoperative complications is provided in Table 2.

**TABLE 2:** Detailed postoperative complications.

Complications (no, %)	*n* = 30
Overall	6 (20.0%)
Complete flap loss	2 (6.7%)
Patient death in ICU (< 48 h)	2 (6.7%)
Partial flap loss	0 (0%)
Soft tissue infection	0 (0%)
Cranioplasty material infection	2 (6.7%)
Seroma	0 (0%)
Hematoma	0 (0%)
Wound dehiscence	0 (0%)
CSF leak	0 (0%)
Exposure of underlying structures	0 (0%)
Donor site complications	0 (0%)

Abbreviations: CSF, cerebrospinal fluid; ICU, intensive care unit.

## 4 Discussion

The aim of the reconstructive surgery of complex scalp defects is to guarantee optimal protection of the brain, obtaining long‐lasting functional results and simultaneously trying to achieve an acceptable aesthetic appearance (Afifi et al. 2010).

Usually, for soft tissue defects with a maximum diameter > 6–8 cm, or 4–5 cm along the hairline, reconstruction requires microsurgical free tissue transfer to provide a stable and vascularized coverage. Despite the size of the soft tissue defect, other indications for microsurgical reconstruction are: bone exposure associated with radiotherapy, the presence of multiple scars on the scalp, meningeal exposure, and CSF leakage (Hussussian and Reece 2002).

In the literature, only a few studies investigate the advantages and disadvantages of the various free flaps and suggest the use of different flaps for each part of the scalp (Sosin et al. 2015).

The most commonly used flap in scalp reconstruction is the LD‐free flap (Afifi et al. 2010; Iblher et al. 2010; Goh et al. 2010; Simunovic et al. 2016). It has an optimal width and thickness that make it particularly appropriate for the reconstruction of very large defects on convex surfaces. Moreover, despite its bulkiness, late atrophy makes the flap thinner over time, thus improving its aesthetic result (McCombe et al. 2002). Finally, the vascular pedicle has a good caliber (average diameter of 2.5 mm) and can be as long as 15 cm (mean length of 9.5 cm) (Hussussian and Reece 2002; Iblher et al. 2010).

The main drawback of the flap is the inability to work simultaneously in two teams due to the proximity of the two surgical fields in the scalp and in the dorsum and the need to change patient positioning during the operation. Even if some articles report the feasibility of the LD flap in a supine position, dissection of the whole muscle in the supine position is more intricate and particularly an arduous task in obese individuals (Schwabegger et al. 2005). Moreover, the majority of the articles report a segmental harvest of the muscle from its anterior border instead of the harvest of whole muscle, which in some cases is required to cover large defects and could be tricky and time‐consuming if the patient is not placed in lateral decubitus. Finally, skin paddle harvest is difficult, especially near the midline, due to the shoulder roll position and represents a shortcoming of this approach (Englar et al. 2020).

The radial fascio‐cutaneous free flap is mainly used for the reconstruction of the frontal region and offers a good aesthetic result in those cases where no loss of bone substance is reported (Horn et al. 2017; Chicarilli, Ariyan, and Cuono 1986). It provides a pedicle of good length and decent caliber but leaves an unsightly result on the donor site.

The ALT flap has a constant anatomy with a long and sizeable pedicle but also has some limits: for example, it can be thinner in skinny people and does not provide good coverage in case of bone deficiency (Wolff 1998). Finally, as a general consideration, perforator and fascio‐cutaneous flaps cannot be used directly over the meninges, because, due to their thinness, they don't guarantee the containment of the spinal fluid, the perfect filling of the bone defect nor robust protection of the underlying cerebral structures.

The VL free flap, although widely used in microsurgical reconstruction of the head‐neck area (Wolff 1998), is rarely used in the reconstruction of the scalp; nevertheless, we used this flap in most of our scalp reconstructions, with or without cranioplasty or bone/meninges replacement, with an uneventful postoperative course and a low complication rate in the majority of cases.

The flap has several advantages: it is a long, strong, and thick muscle capable of covering large surfaces; in case of large bone loss, its thickness provides a proper and robust protection to the brain; it has a long and good‐sized vascular pedicle that can easily reach both the temporal vessels and the facial vessels, as well as the upper thyroid artery and the tributaries of the internal jugular vein; it is characterized by a low donor site morbidity, especially if harvested with a muscle‐sparing technique; the surgical field is distant from the head and the flap can be harvested while operating on the scalp, avoiding the need to change patient positioning throughout the procedure and offering the possibility of a two‐team approach, that, thanks to the simultaneous cooperation of two surgical teams, could shorten the total operative time; it has a robust fascia that can contribute to the reconstruction of the meninges or be positioned above to strengthen them (Nelson, Serletti, and Wu 2010).

In the majority of cases (28 out of 30), VL was harvested as a muscle‐only flap. We believe that the reconstruction with a muscle flap covered with a partial‐thickness skin graft provides a superior aesthetic result, which is further improved by the natural process of atrophy after 6–12 months, if compared with that offered with a musculocutaneous flap. Moreover, the donor site of a musculocutaneous VL flap can be closed primarily only if the skin paddle is up to 8 cm in width (Suárez et al. 2014; Moratin et al. 2023). In case of defects that require larger skin paddles, as the majority of the defects of our series, two options could be considered: harvesting a skin paddle that is equal to the size of the scalp defect and covering the donor site with a skin graft or harvesting a skin paddle that is smaller than the scalp defect and covering the remaining part of the muscle with a skin graft, while closing the donor site by primary intention. In both cases, the additional morbidity of a skin graft is not avoided and the aesthetic outcome is equal or poorer, especially in the case of a flap with the “patchwork” effect of adjacent skin graft and skin paddle (Seyidova, Anderson, and Abood 2021). Only in two cases, we opted for a musculocutaneous flap: in one case the size of the skin paddle was sufficient to entirely cover the cutaneous scalp defect; in the other case the dimension of the scalp defect pushed us to perform a combined “Razor” ALT‐VL musculocutaneous reconstruction to increase the surface coverage of the flap.

In 10 cases, we employed a muscle‐sparing approach for VL flap harvest. This approach relies on the segmental anatomy of the VL muscle, which can be divided into three partitions (superficial, intermediate, and deep) as demonstrated by Toia et al. (Toia et al. 2015), and consists in the elevation of the superficial muscular partition alone, leaving the underlying intermediate and deep partitions intact and therefore reducing the functional damage to the spared muscle components and the related donor site morbidity.

For what concerns the selection of recipient vessels, the superficial temporal artery and vein are considered by other authors the first choice for microsurgical reconstruction of the head and neck. Although some articles describe their feasibility and reliability, in this series, we chose to use temporal vessels only in selected cases of young patients: we employed them as a secondary option in our reconstructive algorithm because they are small in diameter, usually sclerotic, prone to vasospasm and often characterized by tortuous anatomy and an inconstant location (Halvorson et al. 2009). Moreover, the presence of one comitant vein is an additional and well‐known characteristic of temporal vessels, that in our hands could represent a potential drawback: if the only superficial temporal vein is not suitable as the recipient's vein or when a second anastomosis for venous supercharging is needed, or in cases of intra‐operative failure of the venous anastomosis, the absence of other sizeable lifeboat veins in the adjacent region forces to an alternative anastomosis to a vein in the neck, that often requires an interposition graft if the pedicle is not long enough to reach the cervical region, creating further potential for complications and flap failure (Hansen et al. 2007).

Finally, in case of postoperative complications related to the anastomosis, the selection of new recipient vessels in the neck also requires an interposition vein graft, unless a long exceeding pedicle, which is more prone to kinking‐ is prepared and left in the cheek during primary surgery. This drawback requires careful consideration especially in patients with full‐thickness scalp defects, as a flap failure can be life‐threatening.

All these aspects could hinder surgeons who are not used to these vessels (Mattine and Payne 2022; Vicente‐Pardo et al. 2020) and make them less reliable for microvascular‐free tissue transfer.

Conversely, we usually prefer the cervical vessels as the first option in our reconstructive algorithm. We believe that, in our hands, cervical vessels could represent a more convenient alternative if compared to superficial temporal ones for several reasons: they have a constant and reliable anatomy, they give the opportunity to easily select a second vein in the neck if venous supercharging is required and they are distant from the area of trauma or cancer, thus preventing potential complications to the anastomosis related to wound dehiscence or infection in the site of tissue defect.

If compared to the superficial temporal ones, a potential drawback is the greater distance between the site of the microvascular anastomosis and the most distal part of the defect in the scalp, which can lead in cases to an insufficient flap's pedicle length. Usually, this disadvantage can be easily overcome by harvesting a longer flap. In this case, no healthy tissue is removed for flap inset: in fact, the flap is placed over the defect and the vessels are tunneled subcutaneously or passed through a linear incision in the preauricular region.

We found out that the cervical bulging resulting from this maneuver subsides over the first postoperative months due to the physiologic muscle atrophy caused by the flap's denervation, with a final acceptable cosmetic result.

The additional pedicle length required to reach the neck is easily obtained with further proximal dissection of the flap, which usually in our hands takes no more than 30 min. Moreover, in a study published by our group it is demonstrated that, if the flap is harvested with a muscle‐sparing approach, the classical VL pedicle measuring 6–8 cm can be lengthened up to 17 cm, overcoming the majority of the cervical vessels' drawbacks related to a short flap's pedicle (Toia et al. 2015).

However, despite the advantages and drawbacks of each recipient's vessel, the choice often depends mainly on the surgeon's experience and preferences, and what is true for some surgeons, could be not true for others.

In our series, we did not observe any long‐term complications in the cases where cranioplasty was not performed or delayed as we selected patients with non‐weight‐bearing area defects. There was no CSF leak, wound dehiscence, seroma, hematoma, infection, or any kind of complication at the donor site in the thigh region. The only remarkable complication, except for the early reconstructive failure that is mentioned below, was an infection of the prosthetic cranioplasty material that occurred in two patients who underwent immediate bone reconstruction. Despite the replacement of the previous infected cranioplasty material with a new one, the outbreak of infection wasn't exterminated forcing us to remove it after some weeks.

None of the patients complained about the flap's appearance or excessive bulkiness in the scalp or about pain, discomfort, diminished function, or unaesthetic scars at the donor site in the long postoperative time, with a high satisfaction at the 6 months follow‐up.

In our experience, the VL free flap turned out to be a reliable flap for the reconstruction of large complex scalp defects, although we must report that, among 30 cases of reconstruction, we lost two flaps in the early postoperative time (6.7%) and two patients died in the ICU (6.7%): all four cases involved critical patients with ASA score > 4, hemodynamic instability, very low GCS and extensive defects resulting from previous complications of neurosurgical procedure. Therefore, despite an undeniably high rate of overall reconstructive failure accounting for 13.3% of patients, in the ICU subset of patients we used the flap as a life‐salvage procedure for meningeal exposure, due to the high risk of potentially life‐threatening complications associated with this condition, and we considered the flap loss as a direct consequence of the extremely poor conditions of the patients that finally led them to death.

Limitations of this study comprehend its retrospective nature, the relatively small number of included patients, and the lack of an objective scale for the assessment of the aesthetic outcome of our procedures. Further studies with a larger sample size and a longer follow‐up are advisable to obtain statistical and clinical results with stronger significance.

## 5 Conclusions

Reconstruction of complex scalp defects should achieve the goal of protecting the neuro‐cranial structures, ensuring stable coverage, guaranteeing an acceptable aesthetic result, and lowering the incidence of complications in the postoperative period. The gold standard is unanimously represented by the reconstruction through microsurgical flaps that provide well‐vascularized tissue offering the possibility to cover large defects, reducing the incidence of infections, and ensuring good brain protection even without cranioplasty. In our experience, VL flap represents a good reconstructive option thanks to its several advantages: the low morbidity of the donor site, the possibility to harvest the flap without the need to change position, the abundance of muscle to cover large defects, the length and the good characteristics of the vascular pedicle, the nice atrophy in the postoperative period, the ease in harvest technique and the possibility of a muscle‐sparing approach. In our series, the use of this flap allowed us to obtain a low complication rate and turned out to be a valuable alternative for scalp reconstruction that should be added to each surgeon's armamentarium.

## Ethics Statement

The study has been performed in accordance with the principles stated in the World Medical Association Declaration of Helsinki. The study received the approval of the Ethical Committee of the University Hospital “Paolo Giaccone” of Palermo (approval number No. 10—08/04/2024).

## Consent

Patients gave written informed consent to publish the clinical pictures of their cases.

## Conflicts of Interest

The authors declare no conflicts of interest.

## Data Availability

The data that support the findings of this study are available from the corresponding author upon reasonable request.
